# Corticospinal tract: a new hope for the treatment of post-stroke spasticity

**DOI:** 10.1007/s13760-023-02377-w

**Published:** 2023-09-13

**Authors:** Linxing Huang, Lizhen Yi, Huiyuan Huang, Sheng Zhan, Ruixue Chen, Zenghui Yue

**Affiliations:** College of Acupuncture, Massage and Rehabilitation, Hunan University of Chinese Medicine, Changsha, 410208 China

**Keywords:** Post-stroke spasticity, Corticospinal tract, Astrocytes, Axonal remodeling, Functional recovery

## Abstract

Stroke is the third leading cause of death and disability worldwide. Post-stroke spasticity (PSS) is the most common complication of stroke but represents only one of the many manifestations of upper motor neuron syndrome. As an upper motor neuron, the corticospinal tract (CST) is the only direct descending motor pathway that innervates the spinal motor neurons and is closely related to the recovery of limb function in patients with PSS. Therefore, promoting axonal remodeling in the CST may help identify new therapeutic strategies for PSS. In this review, we outline the pathological mechanisms of PSS, specifically their relationship with CST, and therapeutic strategies for axonal regeneration of the CST after stroke. We found it to be closely associated with astroglial scarring produced by astrocyte activation and its secretion of neurotrophic factors, mainly after the onset of cerebral ischemia. We hope that this review offers insight into the relationship between CST and PSS and provides a basis for further studies.

## Introduction

Stroke is the third leading cause of death and disability worldwide [1]. Post-stroke spasticity (PSS) is the most common complication of stroke but is only one of the many manifestations of upper motor neuron syndrome. Over time, PSS continues to develop without effective interventions, and the disease worsens [2]. Twelve months after stroke, 43.2% of survivors develop spasticity [3]. Spasticity is as high as 97% among survivors of chronic stroke with moderate-to-severe dyskinesia [4]; this places a heavy economic burden on patients’ families and wider society. Therefore, more effective therapies are required to promote recovery from PSS.

Treatment of PSS has largely focused on reducing the area of cerebral ischemia and rescuing neurons from damaged areas of the brain; however, the effects of putative neuroprotectants are less pronounced in clinical trials [5]. Several recent clinical studies report that the degree of corticospinal tract (CST) damage correlates with the severity of spasticity in patients with chronic stroke [6–8] and that there is a significant correlation between motor function improvement and CST remodeling in patients with PSS [9]. A few experimental studies have also observed that by destroying the corresponding CST, the upper motor neurons lose control of the spinal cord, causing spasticity of the contralateral limb [10]. While the pathogenesis of PSS is more complex, the more established mechanism is that PSS is a maladaptive manifestation of the loss of supraspinal inhibitory modulation of spinal reflex pathways, which occurs through a functional reorganization at different levels, involving a physiological mechanism of mutual inactivation of motor centers and excitation of peripheral spinal cord segmental neurons; it is one of the syndromes of upper motor neurons [2, 11]. The CST, as an upper motor neuron [12], is the only direct descending motor pathway and is the main pathway innervating spinal motor neurons closely related to the recovery of limb function after stroke [13]. This suggests that CST is closely related to the pathological mechanisms of PSS. Many studies have demonstrated that motor recovery after stroke depends mainly on the plasticity of the damaged lateral primary motor area, evident through stroke patients and experimental animal studies [14–16], or on the homologous CST axon integrity [17–20]. Spasticity is a common disorder that coexists with dyskinesia after stroke [21–24], and it does interfere with motor recovery after stroke as PSS and other dyskinesias are essentially clinical manifestations of abnormal neuroplasticity and manifestations of common processes [25]. Therefore, promoting axonal regeneration of the damaged side of the CST may be a novel avenue for the treatment of post-stroke spasticity; however, only a few studies exist on the specific link between the CST and PSS, the remodeling of CST after stroke, and the recovery promotion of PSS.

This review aimed to provide an overview of the pathophysiological mechanisms of PSS, including the types of spastic hemiparesis, the close correlation between CST and PSS, and the relationship between PSS and motor recovery. We further reviewed the therapeutic strategies to promote axonal regeneration of the CST after stroke. We found that the astroglial scar and its secreted neurotrophic factors, mainly associated with the activation of astrocytes after the onset of cerebral ischemia, are important for remodeling the CST after stroke. This provides potential novel avenues for the treatment of PSS. Finally, we briefly describe the clinical treatment of PSS in cases where the cerebral cortex is severely damaged, and the cortical inputs are not re-established.

## The pathophysiological mechanisms of post-stroke spasticity

### Spastic paresis

Spastic paresis involves two disorders [26, 27]. The first is a muscular disorder promoted by muscle hypo-mobilization in a short position in the context of paresis, in the hours and days after paresis onset, and this genetically mediated, evolving myopathy is called spastic myopathy [28, 29]. The second is a neurological disorder promoted by sensorimotor restriction in the context of paresis and by the muscle disorder itself. It comprises two distinct components, stretch-sensitive paresis and spastic overactivity. The stretch-sensitive paresis is a decreased access of the central command to the agonist, aggravated by antagonist stretch, which mainly affects the agonist [30]. Spastic overactivity, including spastic dystonia, spastic co-contraction, and spasticity, mostly affects antagonists to desired movements [31–33]. Spastic dystonia is an unwanted, involuntary muscle activation at rest in the absence of stretch or voluntary effort; it superimposes spastic myopathy to cause visible, gradually increasing body deformities [34, 35]. Spastic contraction is an unwanted, involuntary antagonist muscle activation during voluntary effort directed to the agonist, aggravated by antagonist stretch; it is primarily due to misdirection of the supraspinal descending drive and contributes to movement amplitude reduction [32, 36]. Spasticity is a form of hyperreflexia, defined by an enhancement of the velocity-dependent responses to phasic stretch, which is detected and measured at rest [23, 32]. With such a strict definition, spasticity is a useful construct for clinicians as a simple marker of this patient population and a clinical parameter quantifiable at the bedside, in contrast, to functionally more important forms of muscle overactivity, provided that a valid and precise measure is used [27, 37, 38]. In addition, spasticity may be mildly correlated with other forms of spastic muscle overactivity as they may all partially reflect both motoneuronal hyperexcitability and spindle responsiveness [32, 39–42]. The three main forms of overactivity share the same motor neuron hyperexcitability as a contributing factor, with all being predominant in the muscles that are more affected by spastic myopathy [26, 27].

PSS generally refers to spasticity as part of the neurological component of spastic paresis [43]. PSS is a complex clinical phenomenon manifested by increased muscle tone and hyperactive reflexes, considered a neurologic problem and an indication of muscle disorder [44]. The stretch reflex consists of afferent nerve fibers, spinal motor neurons, and efferent nerve fibers, whose excitability is regulated primarily by excitatory and inhibitory signals originating downstream from above the spinal cord [45–48]. In healthy subjects, the stretch reflex is mediated by excitatory connections between Ia afferent fibers from muscle spindles and alpha-motor neurons innervating the same muscles from which they arise. Passive stretching of the muscle excites the muscle spindles, causing the Ia fibers to discharge and send input to the alpha-motor neurons via a major monosynaptic pathway. The alpha-motor neurons then send efferent impulses to the muscle, causing it to contract. The rate of muscle tone recorded by surface electromyography (EMG) in normal subjects at rest shows that the stretched muscle does not produce any reflex contraction when a passive muscle stretch is performed. For example, when EMG of the elbow flexors is recorded during forced elbow extension, no stretch reflex is observed in the biceps when passive displacement occurs at a speed typically used in clinical examination of muscle tone (60°–180°/s). The stretch reflex is present only at more than 200° per second. Therefore, the stretch reflex is not responsible for muscle tone in healthy subjects [49]. Muscle tone in healthy subjects is entirely due to biomechanical factors [50].

In contrast to healthy subjects, assessment of the range of displacement velocities of muscle tone in spasticity patients at rest (fully relaxed) revealed a positive linear relationship between EMG activity of the stretched muscle and the speed of stretching. When passive stretching is slower, the stretch reflex tends to be smaller (lower amplitude), and tension can be perceived as relatively normal or simply increased. When the muscle is stretched faster, the stretch reflex increases, and the examiner can detect an increase in muscle tone [50]. Therefore, spasticity is due to an exaggerated stretch reflex [50]. Theoretically, the exaggerated stretch reflex in spasticity patients may be produced by two factors [49]. The first is the increased excitability of muscle spindles, where passive muscle stretching in spastic patients would cause greater activation of spindle afferents than that induced in normal subjects, taking into account the similar speed and amplitude of passive displacement. The second factor is damage to the central nervous system and abnormal output signals from the downstream conduction bundle, leading to excessive reflex activation of alpha-motor neurons [49, 51].

### Post-stroke spasticity and the corticospinal tract

Alpha motor neurons are found primarily in the anterior horn cells (ANC) of the spinal cord, and their axons extend into the lower part of the spinal cord, connecting with lower motor neurons and transmitting motor commands to the muscles [52]. The axons of most alpha-motor neurons are corticospinal tract fibers that extend from the motor cortex of the brain. These fibers pass through various levels of the pathway central nervous system and eventually connect to the lower motor neurons in the spinal cord. Through this connection, alpha-motor neurons transmit motor commands and control signals to lower motor neurons, directly or indirectly controlling muscle movement [11]. Thus, alpha-motor neurons work closely with CST to process motor control [53]. CST is the main descending motor pathway connecting cortical motor areas to spinal cord neurons [12], mainly playing an inhibitory role [54]; it is divided into anterior and lateral parts and belongs to the upper motor neurons, both originating from the cerebral cortex and ending directly or indirectly (via interneurons) in the anterior horn of the spinal cord [11]. The anterior corticospinal tract begins in the ipsilateral hemispheric cortex, and most fibers cross to the contralateral side, section by section, via the anterior white matter connection and enter the contralateral anterior horn. In contrast, a few fibers do not cross the anterior white matter connection and end directly in the ipsilateral anterior horn. The lateral tracts of the corticospinal cord are located in the posterior part of the lateral cord and originate from the cortex of the contralateral cerebral hemisphere (a few fibers also originate from the ipsilateral cerebral cortex) and end in the anterior horn of the local spinal cord. In addition, the CST of the lateral tract contains more than 90% of the fibers present in the CST, with spinal cortical fibers originating from the gray matter of the spinal cord [55–57], which directly reaches the cerebral cortex and runs the length of the spinal cord where it mainly delivers fibers to the limb muscles and the cortical innervation is contralateral, i.e., the left motor cortex controls the right limb [11]. When the stimulus is activated, the cell bodies of the lateral bundle CST (in the primary motor cortex, the upper motor neurons) send a pulse through the bundle, which eventually passes to the anterior horn of the spinal cord, from where it delivers the pulse to the muscle fibers through the alpha-motor neurons. Therefore, CST contains fibers from the upper motor neurons for the synapses of the lower motor neurons [11].

**Fig. 1:**
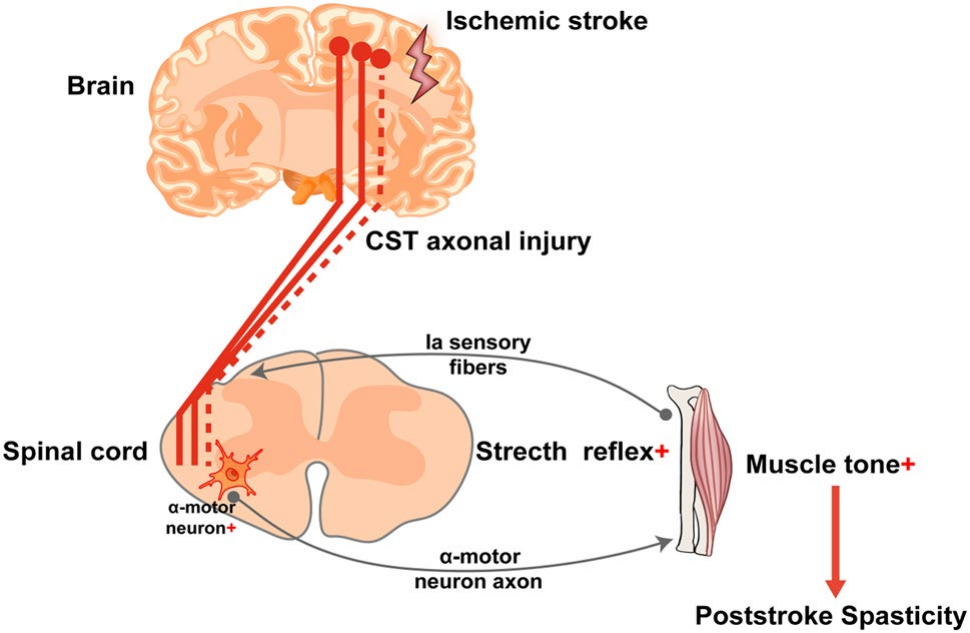
Pathophysiology of post-stroke spasticity (PSS) associated with the corticospinal tract, modified from Wang et al. [44]. The stretch reflex arc is mediated by excitatory connections between Ia afferent fibers from muscle spindles and the alpha-motor neurons innervating the same muscles they arise (indicated by the two gray neuron arrow loops in the figure). When damage to superior motor neurons in the cerebral cortex occurs after ischemic brain injury, i.e., damage to CST axons (red dashed line), resulting in diminished or lost inhibitory control of the CST, abnormal excitation of lower motor neurons-alpha-motor neurons unbalances the excitatory and inhibitory regulation of the spinal retractor reflex by downstream conduction, manifesting hyper-retractor reflexes, and increased muscle tone, producing PSS. ( +): abnormal excitation

Stroke-induced brain injury may affect the integrity and conduction function of the CST [58–61]. First, cerebral ischemia damages the neural axons in the CST. Axons are long protrusions extending from neurons that transmit signals from neurons to target tissues. Ischemic injury may lead to changes such as axon disruption, which interferes with signaling in the CST. In addition, ischemic injury directly affects the upper motor neurons in the CST. These upper motor neurons are located in the motor areas of the cerebral cortex and are responsible for controlling the transmission of motor signals to the spinal cord. Injury to upper motor neurons leads to the loss of their function and affects the transmission of signals from the lower motor neurons. A growing body of experimental evidence supports that the pathological mechanism of PSS may be due to damage to upper motor neurons in the cerebral cortex after stroke, resulting in disinhibition of the CST [11], diminished downward inhibition of upper motor neurons between the cortex and spinal cord, which imbalances the excitatory and inhibitory regulation of downward transmission to the spinal tract reflex, and lower motor neuron-α motor neurons are hyper-reflexively activated, exhibiting a hyper-retentive reflex and increased muscle tone (Fig. 1**)** [2, 11]**.**

## Post-stroke spasticity and motor recovery

Motor recovery after a stroke has various definitions, with “true” motor recovery implying that undamaged brain regions produce commands to the same muscles to produce the same motor patterns [62, 63]. This usually requires restoring or repairing damaged neural tissue, which may occur during a stroke’s acute or subacute phase. However, according to the International Classification of Functioning, Disability, and Health, the motor recovery after stroke is also defined as “improvement in the performance of functional tasks,” i.e., functional recovery. This refers to new motor patterns (different muscles) controlled by alternative brain regions to accomplish the task goals [62–64]. Admittedly, stroke rehabilitation focuses on maximizing and improving the ability to perform such functional tasks [25]. As that neural injury may result in the loss of skilled motor behavior, motor relearning would depend on the reacquisition (recovery) of these basic motor patterns or, in the absence of reacquisition, the adaptation (compensation) of remaining motor elements, or the integration (replacement) of alternative motor elements [65–67].

During full motor recovery, whether hemorrhagic or ischemic, cortical or subcortical, the motor recovery follows a relatively predictable pattern [68]. Brunnstrom [69] described the stereotypical stages of motor recovery: (1) relaxation; (2) appearance of spasticity; (3) increased spasticity by coordinated voluntary movements; (4) onset of motor patterns due to synergism and spasticity decrease; (5) more complex movements and spasticity continue to decrease; (6) spasticity disappears; and (7) complete return to normal function through coordinated voluntary movements. There are three recovery stages: flaccidity, spasticity (emergence, worsening, and reduction, stages 2–5), and recovery (voluntary control without spasticity, stages 6–7). During motor recovery, stroke survivors can progress from one recovery phase to the next at different rates, always in an orderly fashion, without missing any phases. However, recovery may stop in either stage [68, 69]. The classification of these motor recovery phases is widely accepted and used in current clinical practice.

Hyperreflexia and spasticity gradually develop after a stroke. For example, in one study, 87% had detectable spasticity 6 weeks after stroke based on the EMG test results. Increased spasticity was measured on EMG in 92% of patients at 36 weeks post-stroke [70]. It is important to note that spontaneous spasticity reduction is rare; it can evolve and become more severe over time. One study reported spasticity in up to 97% of chronic stroke survivors with moderate and severe motor impairment [24]. The appearance of spasticity, although highly variable [71], usually occurs between 1 and 6 weeks after the initial injury [72]. This implies that the development of spasticity after stroke is related to changes in neuroplasticity within the CNS after the initial injury [31, 32, 46, 47, 72–74]. In chronic stroke survivors with persistent moderate/severe motor impairment, the prevalence of spasticity is increased, and the increased muscle tone significantly predicts overall motor impairment [75, 76], suggesting that the degree of motor impairment after stroke is closely related to the development of spasticity [77–79]. Spasticity is a common disorder that coexists with dyskinesia after stroke [21–24] and interferes with motor recovery after stroke. The development of spasticity is a milestone in the recovery process, and different stages of motor recovery in chronic stroke may reflect different underlying pathophysiology during motor recovery and spasticity [80].

## Implications of promoting CST remodeling for functional recovery in patients with PSS

The degree of CST damage in post-stroke patients is a significant predictor of motor deficits [81], and the integrity of the CST is probably the most important factor affecting the clinical function of patients. CST assessment includes transcranial magnetic stimulation (TMS) and diffusion tensor imaging (DTI) [82, 83]. Motor-evoked potentials (MEP) elicited by TMS provide a quantitative method for assessing the functional integrity of the CST [84, 85]. TMS induces a rapidly changing magnetic field that stimulates cortical neurons and generates induced currents. The induced current depolarizes cortical axons and triggers the MEP at suprathreshold stimulus intensities. MEP is delivered to peripheral muscles via descending pathways, such as the CST and corticobulbar motor pathways [84], providing insight into mechanisms of motor output control [86], which can be used to monitor the clinical progression of stroke recovery [87]. The degree of CST involvement directly correlates with the severity of functional deficits and is inversely related to the degree of neurological recovery [13]. For example, assessing the presence of an MEP in the affected upper limb by TMS [7] and the structural integrity of the CST after stroke by functional MRI showed that the potential for functional recovery in patients with chronic stroke depends on the functional integrity of the CST [7]. In addition, damage assessment to the affected corticospinal tract in patients after stroke by DTI 3D reconstruction showed that the diameter of the affected CST was significantly smaller than that of the healthy side and that patients with more severe damage to the affected CST were more likely to experience spasticity [8]. Importantly, there is a significant correlation between improvement in motor function and CST remodeling in patients with PSS [9], and the recovery of patients with post-stroke dysfunction is largely dependent on homologous CST axon integrity in stroke patients and experimental animals [17–20]. After stroke onset, cortical neurons surviving in the peri-infarct motor cortex undergo axonal sprouting and can restore connections between different brain regions [88]. For example, constraint-induced movement therapy (CIMT), observed by DTI, forces the use of the impaired limb by restraining the unaffected limb by promoting ipsilateral lesion CST remodeling and enhancing the recovery of motor function in rats with ischemic stroke [13]. The above findings confirm that promoting damaged CST remodeling to ensure its integrity positively improves post-stroke spasticity.

## Therapeutic strategies to promote axonal remodeling of the corticospinal tract

**Fig. 2:**
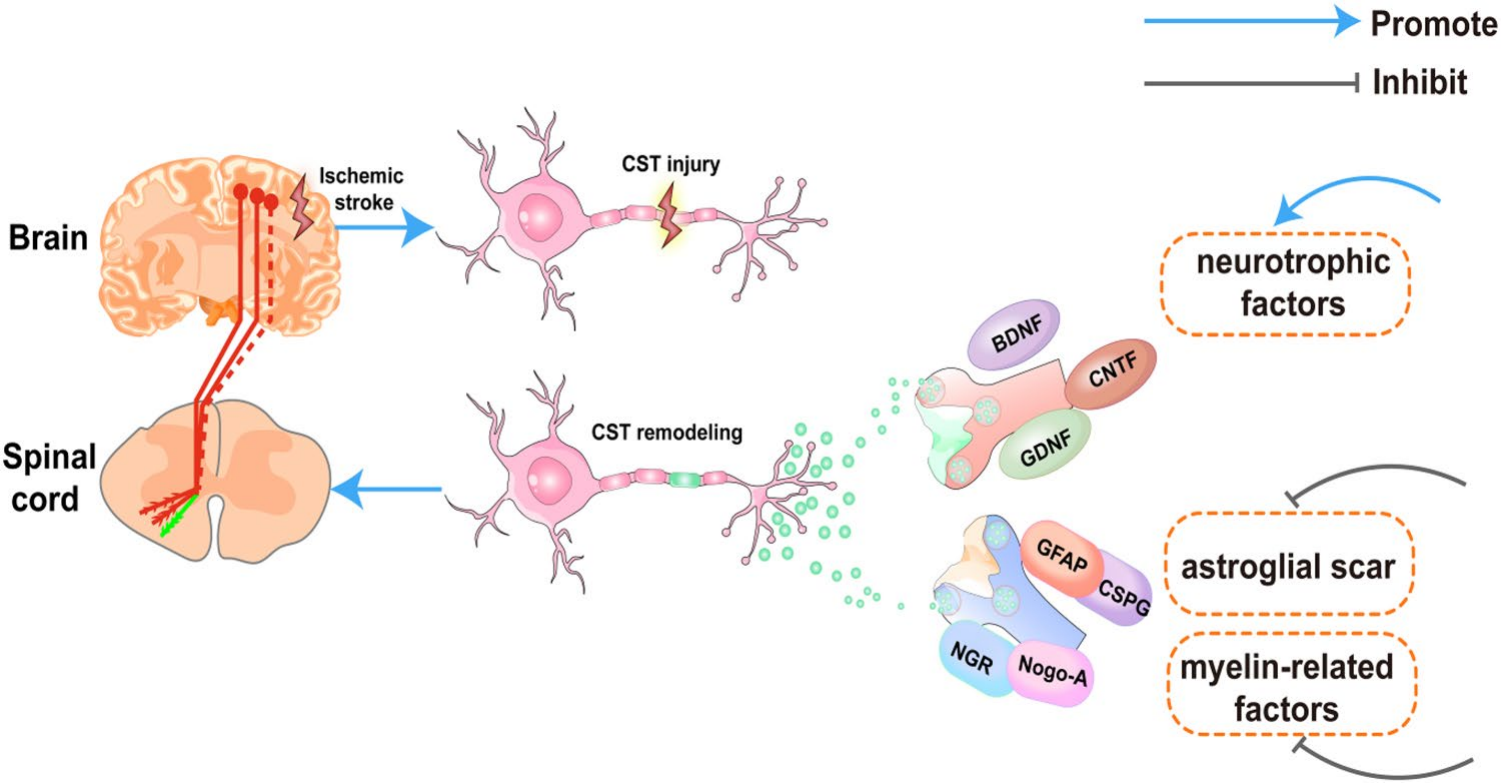
Axonal injury and remodeling of the corticospinal tract (CST) after post-stroke spasticity (PSS) (based on current clinical and laboratory evidence, modified from Liu et al. [61]). Following a stroke, upper motor neurons are impaired, and the descending inhibitory effect of motor neurons (i.e., the CST) between the cortex and spinal cord is attenuated (red and dashed lines represent the CST after injury). This causes abnormal excitation of the spinal cord anterior horn cells, resulting in PSS. To promote axonal remodeling of the CST (indicated by green lines) and restore its function, potential therapeutic strategies include **a** increasing neurotrophins: brain-derived neurotrophic factor (BDNF), glial cell line-derived neurotrophic factor (GDNF), and ciliary neurotrophic factor (CNTF), and **b** reducing the inhibitory factors: glial fibrillary acidic protein (GFAP), chondroitin sulfate (CSPG), myelin inhibitors neurite outgrowth inhibitor (Nogo-A), and nogo–nogo receptor (NGR)

In ischemic brain injury, axonal regeneration of neurons is highly inhibited, severely limiting functional recovery. This is a possible mechanism by which damaged axons cannot grow spontaneously [89], partly because the mature central nervous system axons have a reduced capacity for intrinsic growth and lack external growth stimuli and supportive factors. On the other hand, the central microenvironment has external inhibitors associated with myelin, fibrotic tissue, and astrocyte scars [88, 90]. Thus, targeting axonal remodeling in the side affected by stroke using the treatments above can increase the capability of neuronal outgrowth and reduce the inhibitory factors for axonal outgrowth. None of the strategies specifically target the CST axons in the stroke-impaired spinal gray matter to restore cortical innervation to the denervated spinal motor neurons, the final common pathway of motor control of central nervous system repair after trauma, stroke, or degenerative diseases (Fig. 2) [61, 89].

## Reduction of inhibitory factors

Recent reports suggest that the main reason for difficulties in regeneration after central nervous system injury is that the central nervous system microenvironment is not conducive to nerve regeneration, and glial cells play a vital role in this process. The external inhibitory factor of CST axonal regeneration in the central microenvironment is mainly related to the inhibitory proteins of the astroglial scar, glial fibrillary acidic protein (GFAP), chondroitin sulfate (CSPG), and the myelin inhibitors neurite outgrowth inhibitor (Nogo-A) and nogo–nogo receptor (NGR).

### Inhibition of the astroglial scar

Stroke induces tissue damage in the central nervous system. It activates astrocytes, leading to reactive gliosis, which leads to the formation of astrocytic scars and the production of proteoglycans, inhibiting axon growth and resulting in a physical and biochemical barrier to axon regeneration [91]. Inhibition of astrocyte activation-induced expression of GFAP and CSPG has been shown to promote axonal growth [92, 93].

Astrocyte scars are mainly caused by reactive astrocyte and CSPG constituents, including nerve cancer and phosphate [94, 95]. After central nervous system injury, CSPGs are rapidly upregulated by reactive astrocytes in glial scar tissue [96]. CSPG is also a major inhibitor of axonal regeneration [89, 97]. It secretes CSPGs, which are important in limiting nerve repair by inhibiting axonal growth around the lesion or sprouting spare axonal collateral branches near the lesion [94, 98]. Therefore, inhibition of CSPG activity may be a novel therapeutic strategy to promote axonal regeneration and functional recovery after central nervous system injury [99]. The downregulation of GFAP and CSPG proteins prevents axonal degeneration and improves axonal regeneration [100].

GFAP is a marker of AS activation, and the peak timing of GFAP in the serum of patients with brain injury is earlier, more specific, and more sensitive [101]. It is also a biomarker for nerve injury after stroke [1, 97]. Astrocytes vary with the severity of the injury or distance from the lesion, a dynamic process from swelling and proliferation to glial scar formation. Under physiological conditions, astrocytes cover the entire central nervous system in a continuous and almost non-overlapping manner, and many astrocytes do not express detectable GFAP. When a less severe injury occurs, the expression of GFAP is upregulated and becomes detectable, and the astrocyte cell bodies and processes become hypertrophic [102]. However, the boundaries of each astrocyte are clear and do not overlap. When the injury is more severe, however, the expression of GFAP is upregulated, and the cell bodies of the astrocytes become hypertrophic. Finally, proliferating astrocytes form glial scars between the damaged area and healthy tissue [103]. One study found that CST axons do not regenerate outside the diseased scar, but rather by reducing the expression of the neurite growth inhibitor chondroitin sulfate glycan NG2 and the expression of reactive astroglial marker GFAP, it promotes axonal regeneration of the CST [104].

### Inhibition of myelin-related factors

After cerebral ischemia in adult animals, the inhibitory microenvironment around injured axons is one of the main reasons for the difficulty in central nervous functional repair, resulting in irreversible functional loss [105]. The oligodendrocyte production of myelin-associated inhibitors (MAIs) is an important component of this inhibitory microenvironment. MAIs inhibit development, plasticity, and regeneration after central nervous system injury [106]. Nogo-a and NGR are the major MAI ligands and myelin proteins. After cerebral infarction, over-secretion of Nogo-A by oligodendrocytes is the main factor that inhibits axonal growth [107]. Lindau et al. [108] showed that Nogo-A protein can inhibit the recovery of the structure and function of the CST after ischemic cerebral infarction, and the fibers crossing the midline to half of the denervated spinal cord increased to two-to-three times as much as before, effectively promoting the recovery of nerve function. NGR is a receptor for Nogo-A, a glycosyl-alkyl phospholipid-binding protein located on the surface of neurons. NGR antagonists also contribute to axonal regeneration [109]. Inhibiting the expression of Nogo-A and NGR could maximize functional remodeling of the CST and promote the recovery of neural function [89, 110].

## Increase in neurotrophic factors

Neurotrophins support neuron survival during nerve regeneration, stimulate axon growth, and build lost synapses [111]. Brain-derived neurotrophic factor (BDNF), glial cell line-derived neurotrophic factor (GDNF), and ciliary neurotrophic factor (CNTF) are closely related to CST axon regeneration [112–117].

### BDNF

BDNF is a member of the neurotrophin family of growth factors that play a crucial role in the development of the nervous system while supporting the survival of existing neurons and instigating neurogenesis [118]. Ueno et al. [119] identified BDNF-TrkB signaling essential for CST reorganization. Postsynaptic BDNF triggers morphological changes in TrkB-expressing presynaptic axons to reorganize the network. BDNF is thought to branch laterally in the target region but is not an axonal pathway during development [120]. Our data show that it functions similarly in the damaged adult central nervous system, because TrkB or BDNF knockdown effectively blocks local branching and growth. Still, its effects on remigration and pathfinding of specific regions are limited. By increasing the expression of KCC2 and BDNF in the perilesional cortex and enhancing synaptic plasticity in the denervated cervical spinal cord after cerebral ischemia, the total length of CST fibers sprouting into the denervated cervical spinal cord significantly increased after stroke. A recent study [121] reported CST fibers in the denervated hemispheres could be linked to increased expression levels of BDNF and reduced expression of Nogo-A in the perilesional area.

### GDNF

GDNF, originally isolated from the supernatant of a rat glioma cell line, is a member of the transforming growth factor beta superfamily and is a potent neurotrophic factor in the central and peripheral nervous systems [122]. GDNF expression is upregulated in Ras after stroke [123–127], and RAs-derived GDNF plays an important role in neuronal protection and brain recovery [127]. Recombinant GDNF has a stronger protective effect after middle cerebral artery occlusions [128–130]. However, GDNF deficiency may also increase brain damage [131]. GDNF and its receptors promote axonal growth [132], and it can also promote neurogenesis in the motor neuron system and neuron maturity [133] and mediate neuromuscular connectivity [134]. In the normal brain, GDNF is mainly associated with neurons and is absent or expressed at very low levels in the astrocyte [126, 135]. Neurotrophins support neuronal survival during nerve regeneration, stimulate axonal growth, and establish lost synaptic contacts [111]. GDNF has obvious neuroprotective effects on dopaminergic and cholinergic spinal motor neurons [136]. This stimulates the growth of neural processes [137]. The upregulation of GDNF can promote recovery of neural function after ischemic stroke [138]. GDNF and neurotrophin-3 can rescue cortical and spinal cord neurons from axonal mutation-induced death [113], and overexpression of GDNF and neurotrophin-3 in the sensorimotor cortex near the CST neuronal cell body can significantly increase axonal sprouting [114].

### CNTF

CNTF is a nutrient in the central nervous system astrocytes [112]. CNTF can protect the biceps and has a neurotrophic effect on spinal motor neurons [115]. Studies report that AS provides a bridge for CST axonal regeneration by inhibiting PTEN, a negative regulator of the mammalian target of the rapamycin pathway, and promoting the sprouting of uninjured CST axons to the denervated spinal cord [116, 117], triggering CNTF in local spinal neurons. CNTF may be the trigger factor for CST axonal regeneration, which can transmit cortical signals to the spinal cord to control limb movement and result in remarkable recovery of skilled movement [116, 139]. It has also been found that blocking CNTF can reduce glial scar formation and provide a favorable environment for axonal regeneration [140]. Providing a combination of growth factors at the astrocyte scar border and in the diseased core of the non-nerve tissue stimulates robust regeneration of the proprioceptor spinal axons [141].

## Astrocyte regenerates with CST axon

Reactive astrocytes are closely associated with CST axonal remodeling following stroke. On the one hand, they can improve the central microenvironment to promote the growth of CST axons by inhibiting AS scar-related proteins. However, reactive astrocytes can also enhance the axonal ability to promote the regeneration of CST axons by the derived neurotrophin.

Astrocytes are the most abundant neuroglia in the central nervous system. They contact and communicate with other central nervous system components and are structurally and functionally involved in the nervous system’s normal physiological and pathological reactions [97, 103]. One of stroke’s most important pathological features is the development of reactive astrocytes caused by changes in the central nervous system environment. Reactive astrocytes’ phenotypic and functional characteristics differ in different injury patterns and stages [142]. They repair damaged nerves, inhibit axonal regeneration, and protect the blood–brain barrier while increasing leakage.

Inhibiting CSPG and GFAP in astroglial scars promotes axonal regeneration, and, in more severe cases, reactive astrocytes form permanent glial scars that inhibit axonal regeneration; it is related to poor nerve recovery after stroke [143, 144]. However, numerous studies report that reactive astrocytes produce various neurotrophins, including BDNF, GDNF, and CNTF, to protect neurons after cerebral ischemia [145, 146]. Astrocytes are central to all of these processes.

As mentioned above, the timepoints relating to the development and changes in reactive astrocytes after stroke show that scar tissue plays a dialectical role in nerve fiber regeneration over time and in a specific environment. In the recovery phase after stroke, astrocytic scarring may inhibit axonal regeneration and limit functional recovery; however, it may also protect cells from harmful substances released from the infarct core. Glial scar tissue containing more nutrients in the early stage of axonal regeneration is relatively more beneficial and promotes nerve regeneration [147, 148]. However, old glial scars may be important in inhibiting axonal regeneration [147–150]. The specific relationship between astrocytes and axonal regeneration, including bidirectional effects, should be elucidated in future studies, particularly the relationship between the site and duration of stroke and the degree of astrocyte reaction.

## Clinical treatment of post-stroke spasticity

This review focuses on promoting corticospinal tract axon regeneration in PSS and the mechanisms of related therapeutic strategies. However, the treatment of PSS is more challenging if corticospinal tract axons are not remodeled in the presence of severe cortical damage. Since the CST transmits most of the downstream motor signals from the cortex to the spinal cord, disruption of cortical input can have a major impact on regulating muscle tone and motor control. In situations where cortical inputs to the CST are not adequately restored, alternative therapeutic approaches may be considered to manage spasticity. Many surgical, pharmacological, and therapeutic interventions are used to manage spasticity in clinical settings, individually or in combination [151–154]. Pharmacological interventions [154], such as muscle relaxants (e.g., baclofen [155]) or anti-spasmodic drugs (e.g., botulinum toxin injections [156–158]), may be used to relieve muscle spasms. These drugs act directly on the spinal cord or muscle fibers to reduce muscle hyperexcitability and promote relaxation. Non-pharmacological interventions [159] include acupuncture techniques [44, 160], which are effective in improving spasticity enhancement and motor function; rehabilitation techniques [44, 161], including stretching exercises, passive range of motion exercises, and functional training, which can help control spasticity and neuromuscular electrical stimulation [162, 163], which can reduce spasticity and improve range of motion in patients with PSS. Assistive devices [164]: The use of assistive devices can provide external support and improve functional abilities in individuals with spastic paresis. These devices help with mobility, stability, and compensation for muscle imbalances caused by spasticity. Neurosurgical procedures are considered only for severe spasticity following the failure of pharmacological and/or non-pharmacological management [165].

## Conclusion and outlook

The pathogenesis of PSS is complex. Post-stroke affects the integrity and conduction function of the CST, inducing an imbalance in the excitatory and inhibitory regulation of downward conduction to the spinal tract reflex. Excessive reflex activation of lower motor neurons–alpha-motor neurons may be one of the key pathologies. In the future, more attention should be paid to the connection between the axonal regeneration of the CST, which is responsible for inhibition and spasticity, as well as the physiological mechanisms by which the inactivation of motor centers and excitation of the peripheral, spinal segmental neurons are mutually restricted. In addition, strategies to promote axonal remodeling in the CST include the inhibition of inhibitors in the central microenvironment and an increase in neurotrophins; however, studies report that all surviving corticospinal neurons in both the ipsilateral and contralateral hemispheres are involved in axonal remodeling to compensate for the lost function. Axonal remodeling of which hemisphere is more beneficial to functional recovery after stroke requires further investigation. Finally, this review found that axonal regeneration of the CST after stroke is closely related to astrocytes. However, the specific effects of astrocyte activation on the CST after stroke remain unclear.

In summary, a significant correlation exists between CST remodeling after a stroke and recovery from spasticity. This provides novel avenues to improve PSS and promote axonal regeneration of the CST, enhancing the neuronal reconnection between the motor cortex and spinal cord, releasing the inhibition, and restoring the balance of the spinal cord stretch reflex pathway.

## Data Availability

All data supporting the findings of this study are available within the paper and its Supplementary Information.

## References

[1] Kim JH, Kim SY, Kim B (2021). Prospects of therapeutic target and directions for ischemic stroke. Pharmaceuticals (Basel).

[2] Li S, Francisco GE (2015). New insights into the pathophysiology of post-stroke spasticity. Front Hum Neurosci.

[3] Dorňák T, Justanová M, Konvalinková R (2019). Prevalence and evolution of spasticity in patients suffering from first-ever stroke with carotid origin: a prospective, longitudinal study. Eur J Neurol.

[4] Spa B, Mc A, Ms A, Ct C, Jjdd E (2019). Association of spasticity and motor dysfunction in chronic stroke—ScienceDirect. Ann Phys Rehabil Med.

[5] Schmidt-Pogoda A, Bonberg N, Koecke M (2020). Why most acute stroke studies are positive in animals but not in patients: a systematic comparison of preclinical, early phase, and phase 3 clinical trials of neuroprotective agents. Ann Neurol.

[6] Cho MJ, Yeo SS, Lee SJ, Jang SH (2023). Correlation between spasticity and corticospinal/corticoreticular tract status in stroke patients after early stage. Medicine (Baltimore).

[7] Stinear CM, Barber PA, Smale PR, Coxon JP, Fleming MK, Byblow WD (2007). Functional potential in chronic stroke patients depends on corticospinal tract integrity. Brain.

[8] Liu MY (2019) Magnetic resonance diffusion tensor imaging studies the correlation between corticospinal tract and limb spasticity after cerebral infarction (in chinese). Tongji University, dissertation

[9] Calabrò RS, Naro A, Russo M (2017). Is two better than one? Muscle vibration plus robotic rehabilitation to improve upper limb spasticity and function: a pilot randomized controlled trial. PLoS One.

[10] Zhou T (2021) Establishing a spastic brain injury model in adult SD rat and clinical study of contralateral seventh cervical nerve transfer in the treatment of hand deformity caused by spastic brain injury (in Chinese). Hebei Medical University, phD dissertation

[11] Snell RS (2010). Clinical neuroanatomy[M].

[12] Doughty C, Wang J, Feng W, Hackney D, Pani E, Schlaug G (2016). Detection and predictive value of fractional anisotropy changes of the corticospinal tract in the acute phase of a stroke. Stroke.

[13] Konishi J, Yamada K, Kizu O (2005). MR tractography for the evaluation of functional recovery from lenticulostriate infarcts. Neurology.

[14] Ward NS, Brown MM, Thompson AJ, Frackowiak RS (2003). Neural correlates of outcome after stroke: a cross-sectional fMRI study. Brain.

[15] Baker SN, Zaaimi B, Fisher KM, Edgley SA, Soteropoulos DS (2015). Pathways mediating functional recovery. Prog Brain Res.

[16] Larivière S, Ward NS, Boudrias MH (2018). Disrupted functional network integrity and flexibility after stroke: relation to motor impairments. Neuroimage Clin.

[17] Okabe N, Himi N, Nakamura-Maruyama E (2018). Constraint-induced movement therapy improves efficacy of task-specific training after severe cortical stroke depending on the ipsilesional corticospinal projections. Exp Neurol.

[18] Hu J, Li C, Hua Y (2019). Constrained-induced movement therapy promotes motor function recovery by enhancing the remodeling of ipsilesional corticospinal tract in rats after stroke. Brain Res.

[19] Yarossi M, Patel J, Qiu Q (2019). The association between reorganization of bilateral M1 topography and function in response to early intensive hand focused upper limb rehabilitation following stroke is dependent on ipsilesional corticospinal tract integrity. Front Neurol.

[20] Chen N, Hua Y, Bai Y (2021) Advance in mechanisms of spasticity after stroke[J]. Chin J Rehabil Theor Pract 588–594

[21] Lundström E, Smits A, Terént A, Borg J (2010). Time-course and determinants of spasticity during the first six months following first-ever stroke. J Rehabil Med.

[22] Wissel J, Schelosky LD, Scott J, Christe W, Faiss JH, Mueller J (2010). Early development of spasticity following stroke: a prospective, observational trial. J Neurol.

[23] Marinelli L, Currà A, Trompetto C (2017). Spasticity and spastic dystonia: the two faces of velocity-dependent hypertonia. J Electromyogr Kinesiol.

[24] Pundik S, McCabe J, Skelly M, Tatsuoka C, Daly JJ (2019). Association of spasticity and motor dysfunction in chronic stroke. Ann Phys Rehabil Med.

[25] Li S, Francisco GE, Rymer WZ (2021). A new definition of poststroke spasticity and the interference of spasticity with motor recovery from acute to chronic stages. Neurorehabil Neural Repair.

[26] Baude M, Nielsen JB, Gracies JM (2019). The neurophysiology of deforming spastic paresis: a revised taxonomy. Ann Phys Rehabil Med.

[27] Gracies JM (2015). Coefficients of impairment in deforming spastic paresis. Ann Phys Rehabil Med.

[28] Tabary JC, Tabary C, Tardieu C, Tardieu G, Goldspink G (1972). Physiological and structural changes in the cat's soleus muscle due to immobilization at different lengths by plaster casts. J Physiol.

[29] Giger JM, Bodell PW, Zeng M, Baldwin KM, Haddad F (2009). Rapid muscle atrophy response to unloading: pretranslational processes involving MHC and actin. J Appl Physiol (1985).

[30] Vinti M, Bayle N, Hutin E, Burke D, Gracies JM (2015). Stretch-sensitive paresis and effort perception in hemiparesis. J Neural Transm (Vienna).

[31] Gracies JM (2005). Pathophysiology of spastic paresis. I: Paresis and soft tissue changes. Muscle Nerve.

[32] Gracies JM (2005). Pathophysiology of spastic paresis. II: Emergence of muscle overactivity. Muscle Nerve.

[33] Ansari NN, Naghdi S, Moammeri H, Jalaie S (2006). Ashworth Scales are unreliable for the assessment of muscle spasticity. Physiother Theory Pract.

[34] Phillips CG (1987). The cerebral control of movement. Electroencephalogr Clin Neurophysiol Suppl.

[35] Laplane D, Talairach J, Meininger V, Bancaud J, Bouchareine A (1977). Motor consequences of motor area ablations in man. J Neurol Sci.

[36] Vinti M, Couillandre A, Hausselle J (2013). Influence of effort intensity and gastrocnemius stretch on co-contraction and torque production in the healthy and paretic ankle. Clin Neurophysiol.

[37] Gracies JM, Burke K, Clegg NJ (2010). Reliability of the Tardieu Scale for assessing spasticity in children with cerebral palsy. Arch Phys Med Rehabil.

[38] Gracies JM, Marosszeky JE, Renton R, Sandanam J, Gandevia SC, Burke D (2000). Short-term effects of dynamic lycra splints on upper limb in hemiplegic patients. Arch Phys Med Rehabil.

[39] Gorassini MA, Knash ME, Harvey PJ, Bennett DJ, Yang JF (2004). Role of motoneurons in the generation of muscle spasms after spinal cord injury. Brain.

[40] D'Amico JM, Condliffe EG, Martins KJ, Bennett DJ, Gorassini MA (2014). Recovery of neuronal and network excitability after spinal cord injury and implications for spasticity. Front Integr Neurosci.

[41] D'Amico JM, Murray KC, Li Y (2013). Constitutively active 5-HT2/α1 receptors facilitate muscle spasms after human spinal cord injury. J Neurophysiol.

[42] Gioux M, Petit J (1993). Effects of immobilizing the cat peroneus longus muscle on the activity of its own spindles. J Appl Physiol (1985).

[43] Lackritz H, Parmet Y, Frenkel-Toledo S (2021). Effect of post-stroke spasticity on voluntary movement of the upper limb. J Neuroeng Rehabil.

[44] Wang JX, Fidimanantsoa OL, Ma LX (2023). New insights into acupuncture techniques for poststroke spasticity. Front Public Health.

[45] Young RR (1994). Spasticity: a review. Neurology.

[46] Mukherjee A, Chakravarty A (2010). Spasticity mechanisms—for the clinician. Front Neurol.

[47] Burke D, Wissel J, Donnan GA (2013). Pathophysiology of spasticity in stroke. Neurology.

[48] Heckmann CJ, Gorassini MA, Bennett DJ (2005). Persistent inward currents in motoneuron dendrites: implications for motor output. Muscle Nerve.

[49] Trompetto C, Marinelli L, Mori L (2014). Pathophysiology of spasticity: implications for neurorehabilitation. Biomed Res Int.

[50] Thilmann AF, Fellows SJ, Garms E (1991). The mechanism of spastic muscle hypertonus. Variation in reflex gain over the time course of spasticity. Brain.

[51] Hu X, Suresh NL, Chardon MK, Rymer WZ (2015). Contributions of motoneuron hyperexcitability to clinical spasticity in hemispheric stroke survivors. Clin Neurophysiol.

[52] Biller J (2021). Therapy in neurology. Neurol Clin.

[53] Lemon RN (2008). Descending pathways in motor control. Annu Rev Neurosci.

[54] Jang SH (2009). The role of the corticospinal tract in motor recovery in patients with a stroke: a review. NeuroRehabilitation.

[55] York DH (1987). Review of descending motor pathways involved with transcranial stimulation. Neurosurgery.

[56] Davidoff RA (1990). The pyramidal tract. Neurology.

[57] Canedo A (1997). Primary motor cortex influences on the descending and ascending systems. Prog Neurobiol.

[58] Qing L, Lin L, Hu S (2016). The relation between injury of corticospinal tract and motor function of stroke patients using MR-diffusion tensor imaging[J]. J Rehabil Med.

[59] Bigourdan A, Munsch F, Coupé P (2016). Early fiber number ratio is a surrogate of corticospinal tract integrity and predicts motor recovery after stroke[J]. Stroke.

[60] Zhu ZL, Shen TY, Li XX (2022). Progress of researches on involvement of corticospinal tract in the effect of acupuncture on improvement of post-stroke motor dysfunction[J]. Zhen ci yan jiu = Acupuncture Research.

[61] Liu Z, Xin H, Chopp M (2021). Axonal remodeling of the corticospinal tract during neurological recovery after stroke. Neural Regen Res.

[62] Jones TA (2017). Motor compensation and its effects on neural reorganization after stroke. Nat Rev Neurosci.

[63] Bernhardt J, Hayward KS, Kwakkel G (2017). Agreed definitions and a shared vision for new standards in stroke recovery research: the stroke recovery and rehabilitation roundtable taskforce. Neurorehabil Neural Repair.

[64] Levin MF, Kleim JA, Wolf SL (2009). What do motor "recovery" and "compensation" mean in patients following stroke. Neurorehabil Neural Repair.

[65] Margolis JF, Christina RW (1981). A test of Schmidt's schema theory of discrete motor skill learning. Res Q Exerc Sport.

[66] Information, natural laws, and self-assembly of rhythmic movement. Information, natural laws, and self-assembly of rhythmic movement

[67] Higgins S (1991). Motor skill acquisition. Phys Ther.

[68] Twitchell TE (1951). The restoration of motor function following hemiplegia in man. Brain.

[69] Brunnstrom S (1966). Motor testing procedures in hemiplegia: based on sequential recovery stages. Phys Ther.

[70] Malhotra S, Pandyan AD, Rosewilliam S, Roffe C, Hermens H (2011). Spasticity and contractures at the wrist after stroke: time course of development and their association with functional recovery of the upper limb. Clin Rehabil.

[71] Ward AB (2012). A literature review of the pathophysiology and onset of post-stroke spasticity. Eur J Neurol.

[72] Balakrishnan S, Ward AB (2013). The diagnosis and management of adults with spasticity. Handb Clin Neurol.

[73] Nudo RJ (2006). Mechanisms for recovery of motor function following cortical damage. Curr Opin Neurobiol.

[74] Nielsen JB, Crone C, Hultborn H (2007). The spinal pathophysiology of spasticity–from a basic science point of view. Acta Physiol (Oxf).

[75] Plantin J, Pennati GV, Roca P (2019). Quantitative assessment of hand spasticity after stroke: imaging correlates and impact on motor recovery. Front Neurol.

[76] Sunnerhagen KS, Opheim A, Alt MM (2019). Onset, time course and prediction of spasticity after stroke or traumatic brain injury. Ann Phys Rehabil Med.

[77] Balakrishnan S, Ward AB (2013). The diagnosis and management of adults with spasticity—ScienceDirect. Handb Clin Neurol.

[78] Opheim A, Danielsson A, Alt Murphy M, Persson HC, Sunnerhagen KS (2015). Early prediction of long-term upper limb spasticity after stroke: part of the SALGOT study. Neurology.

[79] Wissel J, Verrier M, Simpson DM (2015). Post-stroke spasticity: predictors of early development and considerations for therapeutic intervention. PM R.

[80] Li S (2017). Spasticity, motor recovery, and neural plasticity after stroke. Front Neurol.

[81] Zhu LL, Lindenberg R, Alexander MP, Schlaug G (2010). Lesion load of the corticospinal tract predicts motor impairment in chronic stroke. Stroke.

[82] Jang SH (2013). Motor recovery by improvement of limb-kinetic apraxia in a chronic stroke patient. NeuroRehabilitation.

[83] Potter-Baker KA, Varnerin NM, Cunningham DA (2016). Influence of corticospinal tracts from higher order motor cortices on recruitment curve properties in stroke. Front Neurosci.

[84] Groppa S, Oliviero A, Eisen A (2012). A practical guide to diagnostic transcranial magnetic stimulation: report of an IFCN committee. Clin Neurophysiol.

[85] Okamoto Y, Ishii D, Yamamoto S (2021). Relationship between motor function, DTI, and neurophysiological parameters in patients with stroke in the recovery rehabilitation unit. J Stroke Cerebrovasc Dis.

[86] Bestmann S, Krakauer JW (2015). The uses and interpretations of the motor-evoked potential for understanding behaviour. Exp Brain Res.

[87] Cakar E, Akyuz G, Durmus O (2016). The relationships of motor-evoked potentials to hand dexterity, motor function, and spasticity in chronic stroke patients: a transcranial magnetic stimulation study. Acta Neurol Belg.

[88] Liu Z, Li Y, Zhang ZG (2010). Bone marrow stromal cells enhance inter- and intracortical axonal connections after ischemic stroke in adult rats. J Cereb Blood Flow Metab.

[89] Anderson MA, Burda JE, Ren Y (2016). Astrocyte scar formation aids central nervous system axon regeneration. Nature.

[90] Neuroscience JR (2006). A neuronal receptor for botulinum toxin. Science.

[91] Yiu G, He Z (2006). Glial inhibition of CNS axon regeneration. Nat Rev Neurosci.

[92] Liu Z, Li Y, Cui Y (2014). Beneficial effects of gfap/vimentin reactive astrocytes for axonal remodeling and motor behavioral recovery in mice after stroke. Glia.

[93] Xu X, Bass B, McKillop WM (2018). Sox9 knockout mice have improved recovery following stroke. Exp Neurol.

[94] Silver J, Miller JH (2004). Regeneration beyond the glial scar. Nat Rev Neurosci.

[95] Sofroniew MV (2009). Molecular dissection of reactive astrogliosis and glial scar formation. Trends Neurosci.

[96] Sharma K, Selzer ME, Li S (2012). Scar-mediated inhibition and CSPG receptors in the CNS. Exp Neurol.

[97] Liu Z, Chopp M (2016). Astrocytes, therapeutic targets for neuroprotection and neurorestoration in ischemic stroke. Prog Neurobiol.

[98] Bradbury EJ, Moon LD, Popat RJ (2002). Chondroitinase ABC promotes functional recovery after spinal cord injury. Nature.

[99] Li L, Ni L, Eugenin EA, Heary RF, Elkabes S (2019). Toll-like receptor 9 antagonism modulates astrocyte function and preserves proximal axons following spinal cord injury. Brain Behav Immun.

[100] Ding Y, Yan Q, Ruan JW (2011). Bone marrow mesenchymal stem cells and electroacupuncture downregulate the inhibitor molecules and promote the axonal regeneration in the transected spinal cord of rats. Cell Transplant.

[101] Senn R, Elkind MS, Montaner J, Christ-Crain M, Katan M (2014). Potential role of blood biomarkers in the management of nontraumatic intracerebral hemorrhage. Cerebrovasc Dis.

[102] Kajihara H, Tsutsumi E, Kinoshita A, Nakano J, Takagi K, Takeo S (2001). Activated astrocytes with glycogen accumulation in ischemic penumbra during the early stage of brain infarction: immunohistochemical and electron microscopic studies. Brain Res.

[103] Shen XY, Gao ZK, Han Y, Yuan M, Guo YS, Bi X (2021). Activation and role of astrocytes in ischemic stroke. Front Cell Neurosci.

[104] Chen J, Wu J, Apostolova I (2007). Adeno-associated virus-mediated L1 expression promotes functional recovery after spinal cord injury. Brain.

[105] Fawcett JW (2018). The paper that restarted modern central nervous system axon regeneration research. Trends Neurosci.

[106] Boghdadi AG, Teo L, Bourne JA (2017). The involvement of the myelin-associated inhibitors and their receptors in CNS plasticity and injury. Mol Neurobiol.

[107] Wiessner C, Bareyre FM, Allegrini PR (2003). Anti-Nogo-A antibody infusion 24 hours after experimental stroke improved behavioral outcome and corticospinal plasticity in normotensive and spontaneously hypertensive rats. J Cereb Blood Flow Metab.

[108] Lindau NT, Bänninger BJ, Gullo M (2014). Rewiring of the corticospinal tract in the adult rat after unilateral stroke and anti-Nogo-A therapy. Brain.

[109] Teng FY, Tang BL (2005). Why do Nogo/Nogo-66 receptor gene knockouts result in inferior regeneration compared to treatment with neutralizing agents. J Neurochem.

[110] Geoffroy CG, Lorenzana AO, Kwan JP (2015). Effects of PTEN and Nogo codeletion on corticospinal axon sprouting and regeneration in mice. J Neurosci.

[111] Markosyan V, Safiullov Z, Izmailov A, Fadeev F, Islamov R (2020). Preventive triple gene therapy reduces the negative consequences of ischemia-induced brain injury after modelling stroke in a rat. Int J Mol Sci.

[112] Louis JC, Magal E, Takayama S, Varon S (1993). CNTF protection of oligodendrocytes against natural and tumor necrosis factor-induced death. Science.

[113] Giehl KM, Schütte A, Mestres P, Yan Q (1998). The survival-promoting effect of glial cell line-derived neurotrophic factor on axotomized corticospinal neurons in vivo is mediated by an endogenous brain-derived neurotrophic factor mechanism. J Neurosci.

[114] Zhou L, Shine HD (2003). Neurotrophic factors expressed in both cortex and spinal cord induce axonal plasticity after spinal cord injury. J Neurosci Res.

[115] Vergara C, Ramirez B (2004). CNTF, a pleiotropic cytokine: emphasis on its myotrophic role. Brain Res Brain Res Rev.

[116] Zukor K, Belin S, Wang C, Keelan N, Wang X, He Z (2013). Short hairpin RNA against PTEN enhances regenerative growth of corticospinal tract axons after spinal cord injury. J Neurosci.

[117] Leibinger M, Zeitler C, Gobrecht P, Andreadaki A, Gisselmann G, Fischer D (2021). Transneuronal delivery of hyper-interleukin-6 enables functional recovery after severe spinal cord injury in mice. Nat Commun.

[118] Eyileten C, Sharif L, Wicik Z (2021). The relation of the brain-derived neurotrophic factor with MicroRNAs in neurodegenerative diseases and ischemic stroke. Mol Neurobiol.

[119] Ueno M, Hayano Y, Nakagawa H, Yamashita T (2012). Intraspinal rewiring of the corticospinal tract requires target-derived brain-derived neurotrophic factor and compensates lost function after brain injury. Brain.

[120] Cohen-Cory S, Kidane AH, Shirkey NJ, Marshak S (2010). Brain-derived neurotrophic factor and the development of structural neuronal connectivity. Dev Neurobiol.

[121] Cheng X, Wang H, Liu C (2019). Dl-3-n-butylphthalide promotes remyelination process in cerebral white matter in rats subjected to ischemic stroke. Brain Res.

[122] Allen SJ, Watson JJ, Shoemark DK, Barua NU, Patel NK (2013). GDNF, NGF and BDNF as therapeutic options for neurodegeneration. Pharmacol Ther.

[123] Sakurai M, Hayashi T, Abe K (1999). Induction of glial cell line-derived neurotrophic factor and c-ret porto-oncogene-like immunoreactivity in rabbit spinal cord after transient ischemia. Neurosci Lett.

[124] Wei GW, Wu GC, Cao XD (2000). Dynamic expression of glial cell line-derived neurotrophic factor after cerebral ischemia. NeuroReport.

[125] Arvidsson A, Kokaia Z, Airaksinen MS, Saarma M, Lindvall O (2001). Stroke induces widespread changes of gene expression for glial cell line-derived neurotrophic factor family receptors in the adult rat brain. Neuroscience.

[126] Miyazaki H, Nagashima K, Okuma Y, Nomura Y (2001). Expression of glial cell line-derived neurotrophic factor induced by transient forebrain ischemia in rats. Brain Res.

[127] Zhang Z, Zhang N, Ding S (2022). Glial cell line derived neurotrophic factor in brain repair after focal ischemic stroke. Neural Regen Res.

[128] Kobayashi T, Ahlenius H, Thored P, Kobayashi R, Kokaia Z, Lindvall O (2006). Intracerebral infusion of glial cell line—derived. Stroke.

[129] Nosheny RL, Bachis A, Aden SA, Bernardi MAD, Mocchetti I (2006). Intrastriatal administration of human immunodeficiency virus-1 glycoprotein 120 reduces glial cell-line derived neurotrophic factor levels and causes apoptosis in the substantia nigra. J Neurobiol.

[130] Yoshifumi H, Osamu H (2006). Intravenous administration of glial cell line-derived neurotrophic factor gene-modified human mesenchymal stem cells protects against injury in a cerebral ischemia model in the adult rat. J Neurosci Res.

[131] Zhang N, Zhang Z, He R, Li H, Ding S (2020). GLAST-CreERT2 mediated deletion of GDNF increases brain damage and exacerbates long-term stroke outcomes after focal ischemic stroke in mouse model. Glia.

[132] Hellmich HL, Kos L, Cho ES, Mahon KA, Zimmer A (1996). Embryonic expression of glial cell-line derived neurotrophic factor (GDNF) suggests multiple developmental roles in neural differentiation and epithelial-mesenchymal interactions. Mech Dev.

[133] Uesaka T, Nagashimada M, Enomoto H (2013). GDNF signaling levels control migration and neuronal differentiation of enteric ganglion precursors. J Neurosci.

[134] Cortés D, Robledo-Arratia Y, Hernández-Martínez R, Escobedo-Ávila I, Bargas J, Velasco I (2016). Transgenic GDNF positively influences proliferation, differentiation, maturation and survival of motor neurons produced from mouse embryonic stem cells. Front Cell Neurosci.

[135] Kuric E, Wieloch T, Ruscher K (2013). Dopamine receptor activation increases glial cell line-derived neurotrophic factor in experimental stroke. Exp Neurol.

[136] Cheng H, Wu JP, Tzeng SF (2002). Neuroprotection of glial cell line-derived neurotrophic factor in damaged spinal cords following contusive injury. J Neurosci Res.

[137] Iannotti C, Li H, Yan P, Lu X, Wirthlin L, Xu XM (2003). Glial cell line-derived neurotrophic factor-enriched bridging transplants promote propriospinal axonal regeneration and enhance myelination after spinal cord injury. Exp Neurol.

[138] Liu Y, Zhao Y, Min Y (2021). Effects and mechanisms of bone marrow mesenchymal stem cell transplantation for treatment of ischemic stroke in hypertensive rats. Int J Stem Cells.

[139] Jin D, Liu Y, Sun F, Wang X, Liu X, He Z (2015). Restoration of skilled locomotion by sprouting corticospinal axons induced by co-deletion of PTEN and SOCS3. Nat Commun.

[140] Ishii K, Nakamura M, Dai H (2006). Neutralization of ciliary neurotrophic factor reduces astrocyte production from transplanted neural stem cells and promotes regeneration of corticospinal tract fibers in spinal cord injury. J Neurosci Res.

[141] Anderson MA, Shea TM (2018). Required growth facilitators propel axon regeneration across complete spinal cord injury. Nature.

[142] Li L, Zhou J, Han L (2022). The specific role of reactive astrocytes in stroke. Front Cell Neurosci.

[143] Buss A, Brook GA, Kakulas B (2004). Gradual loss of myelin and formation of an astrocytic scar during Wallerian degeneration in the human spinal cord. Brain.

[144] Escartin C, Guillemaud O, Carrillo-de Sauvage MA (2019). Questions and (some) answers on reactive astrocytes. Glia.

[145] Pöyhönen S, Er S, Domanskyi A, Airavaara M (2019). Effects of neurotrophic factors in glial cells in the central nervous system: expression and properties in neurodegeneration and injury. Front Physiol.

[146] Duarte EP, Curcio M, Canzoniero LM, Duarte CB (2012). Neuroprotection by GDNF in the ischemic brain. Growth Factors.

[147] Li X, Yang B, Xiao Z (2018). Comparison of subacute and chronic scar tissues after complete spinal cord transection. Exp Neurol.

[148] Hara M, Kobayakawa K, Ohkawa Y (2017). Interaction of reactive astrocytes with type I collagen induces astrocytic scar formation through the integrin-*N*-cadherin pathway after spinal cord injury. Nat Med.

[149] Schäfer M, Tegeder I (2018). NG2/CSPG4 and progranulin in the posttraumatic glial scar. Matrix Biol.

[150] Silver J (2016). The glial scar is more than just astrocytes. Exp Neurol.

[151] Lindsay C, Kouzouna A, Simcox C, Pandyan AD (2016). Pharmacological interventions other than botulinum toxin for spasticity after stroke. Cochrane Database Syst Rev.

[152] McIntyre A, Lee T, Janzen S, Mays R, Mehta S, Teasell R (2012). Systematic review of the effectiveness of pharmacological interventions in the treatment of spasticity of the hemiparetic lower extremity more than six months post stroke. Top Stroke Rehabil.

[153] Otero-Romero S, Sastre-Garriga J, Comi G (2016). Pharmacological management of spasticity in multiple sclerosis: systematic review and consensus paper. Mult Scler.

[154] Yelnik AP, Simon O, Bensmail D (2009). Drug treatments for spasticity. Ann Phys Rehabil Med.

[155] Nishimura F, Park YS, Motoyama Y (2019). Intrathecal baclofen therapy for spasticity in post-stroke patients. Surg Cerebral Stroke.

[156] Sheean G (2009). Botulinum toxin should be first-line treatment for poststroke spasticity. J Neurol Neurosurg Psychiatry.

[157] Kakuda W, Abo M, Momosaki R (2012). Combined therapeutic application of botulinum toxin type A, low-frequency rTMS, and intensive occupational therapy for post-stroke spastic upper limb hemiparesis. Eur J Phys Rehabil Med.

[158] Kinnear BZ, Lannin NA, Cusick A, Harvey LA, Rawicki B (2014). Rehabilitation therapies after botulinum toxin-A injection to manage limb spasticity: a systematic review. Phys Ther.

[159] Khan F, Amatya B, Bensmail D, Yelnik A (2019). Non-pharmacological interventions for spasticity in adults: an overview of systematic reviews. Ann Phys Rehabil Med.

[160] Lim SM, Yoo J, Lee E (2015). Acupuncture for spasticity after stroke: a systematic review and meta-analysis of randomized controlled trials. Evid Based Complement Alternat Med.

[161] Babazadeh-Zavieh SS, Ansari NN, Ghotbi N (2022). Effects of dry needling plus exercise therapy on post-stroke spasticity and motor function: a case report[J]. Complement Ther Clin Pract.

[162] Brusola G, Garcia E, Albosta M, Daly A, Kafes K, Furtado M (2023). Effectiveness of physical therapy interventions on post-stroke spasticity: an umbrella review. NeuroRehabilitation.

[163] Stein C, Fritsch CG, Robinson C, Sbruzzi G, Plentz RD (2015). Effects of electrical stimulation in spastic muscles after stroke: systematic review and meta-analysis of randomized controlled trials. Stroke.

[164] Seim C, Chen B, Han C, Vacek D, Lowber A, Lansberg M, Okamura AM (2023) Daily vibrotactile stimulation from a wearable device exhibits equal or greater spasticity relief than botulinum toxin in stroke. Arch Phys Med Rehabil S0003-9993(23)00238-1 10.1016/j.apmr.2023.03.031 PMC11326884 37149017

[165] Lazorthes Y, Sol JC, Sallerin B, Verdié JC (2002). The surgical management of spasticity. Eur J Neurol.

